# Association of Menopausal Vasomotor Symptoms With Increased Bone Turnover During the Menopausal Transition

**DOI:** 10.1002/jbmr.259

**Published:** 2010-09-27

**Authors:** Carolyn J Crandall, Chi-Hong Tseng, Sybil L Crawford, Rebecca C Thurston, Ellen B Gold, Janet M Johnston, Gail A Greendale

**Affiliations:** 1 Department of Medicine, University of California Los AngelesLos Angeles, CA, USA; 2 Department of Medicine, University of Massachusetts Medical SchoolWorcester, MA, USA; 3 Departments of Psychiatry and Epidemiology, University of Pittsburgh School of MedicinePittsburgh, PA, USA; 4 Department of Public Health Sciences, University of California DavisDavis, CA, USA; 5 Department of Health Sciences, University of Alaska AnchorageAnchorage, AL, USA

**Keywords:** HOT FLASHES, VASOMOTOR SYMPTOMS, BONE TURNOVER, URINARY *N*-TELOPEPTIDE, NTX

## Abstract

The purpose of this study was to determine the longitudinal association between menopausal vasomotor symptoms (VMS) and urinary *N*-telopeptide level (NTX) according to menopausal stage. We analyzed data from 2283 participants of the Study of Women's Health Across the Nation, a longitudinal community-based cohort study of women aged 42 to 52 years at baseline. At baseline and annually through follow-up visit 8, participants provided questionnaire data, urine samples, serum samples, and anthropometric measurements. Using multivariable repeated-measures mixed models, we examined associations between annually assessed VMS frequency and annual NTX measurements. Our results show that mean adjusted NTX was 1.94 nM of bone collagen equivalents (BCE)/mM of creatinine higher among early perimenopausal women with any VMS than among early perimenopausal women with no VMS (*p* < .0001). Mean adjusted NTX was 2.44 nM BCE/mM of creatinine higher among late perimenopausal women with any VMS than among late perimenopausal women with no VMS (*p* = .03). Among premenopausal women, VMS frequency was not significantly associated with NTX level. When NTX values among women with frequent VMS (≥6 days in past 2 weeks) were expressed as percentages of NTX values among women without frequent VMS, the differences were 3% for premenopausal women, 9% for early perimenopausal women, 7% for late perimenopausal women, and 4% for postmenopausal women. Adjustment for serum follicle-stimulating hormone (FSH) level greatly reduced the magnitudes of associations between VMS and NTX level. We conclude that among early perimenopausal and late perimenopausal women, those with VMS had higher bone turnover than those without VMS. Prior to the final menstrual period, VMS may be a marker for risk of adverse bone health. © 2011 American Society for Bone and Mineral Research.

## Introduction

Vasomotor symptoms (hot flashes and/or night sweats; VMS) occur in approximately 50% to 70% of peri- and postmenopausal women.(1–7) VMS prevalence increases sharply in the 2 years *before* the final menstrual period.(6,7) Approximately 35% to 50% of pre- and perimenopausal women report having VMS.(1–5,7) Thus VMS are not uncommon prior to the final menstrual period.

In studies of midlife women, we and others have found that mean bone mineral density (BMD) is lower among women with VMS than among women without VMS.(8–11) One possible reason for this inverse association between VMS and BMD is that VMS may be associated with increased bone turnover, in turn, leading to decreased BMD.

An association of VMS with increased bone turnover in midlife women might be expected on the basis of prior studies linking cortisol and sympathetic nervous system activation to both hot flashes and BMD. Serum cortisol and serum epinephrine may increase in association with hot flashes,(12–14) and systolic blood pressure may be higher among women with hot flashes than among women without hot flashes.(15) Also, brain norepinephrine metabolites increase during hot flashes.(16)

Sustained or frequent elevations in serum cortisol and/or serum epinephrine owing to their relations with hot flashes may have adverse effects on bone health. For example, medication-induced blockage of the sympathetic nervous system (by beta blockers) has favorable effects on trabecular microarchitecture and cortical width of the femur, as well as hip and lumbar BMD, in postmenopausal women.(17) In addition, the majority of case-control studies of BMD that have included women have reported higher BMD among users compared with nonusers, of beta blockers.(17–19) Beta-blocker administration increases BMD in mice.(19) Conversely, administration of beta-agonist medications has deleterious effects on trabecular bone architecture and accelerates bone resorption in rats(20) and decreases BMD in mice.(21) An additional theory for the association between VMS and BMD could be interindividual differentials in estrogen “sensitivity” at the menopause transition; a higher sensitivity to the reduction in estrogen levels may underlie certain women having both frequent VMS and greater bone loss.

An association of VMS with increased bone turnover [urinary *N*-telopeptide level (NTX)] was found in one cross-sectional study of infertile premenopausal women,(22) but the association has not been studied prospectively over the menopause transition. Given the substantial prevalence of VMS even among women who have not yet had their final menstrual period, an association of VMS with adverse indicators of bone health could affect many women. We hypothesized that the presence of VMS would be related to higher levels of urinary *N*-telopeptide among midlife women even prior to the postmenopausal stage. To address this hypothesis, we used longitudinal data collected from baseline and eight annual follow-up visits in the Study of Women's Health Across the Nation (SWAN), a large, prospective, community-based study of the menopausal transition.

## Methods

### The Study of Women's Health Across the Nation (SWAN) and the SWAN bone study

To determine the association between BMD and urinary NTX level, we used data from baseline through annual follow-up visit 8 from SWAN, a multisite, longitudinal, community-based cohort study of 3302 women. At baseline, SWAN participants were aged 42 to 52 years and premenopausal or early perimenopausal with an intact uterus and one or more ovaries, were not pregnant or lactating, and were not using exogenous reproductive hormones.(23) (Please see below for a definitions of menopausal stages.) Each of the seven study sites enrolled white women in addition to women of one other site-specific, self-identified racial/ethnic group: African-American women (Boston, Detroit area, Chicago, and Pittsburgh), Japanese women (Los Angeles), Hispanic women (New Jersey), and Chinese women (Oakland, CA). SWAN participants completed questionnaires, provided a morning urine sample, and underwent fasting blood sampling annually. If annual phlebotomy could not be performed in the early follicular phase (days 2 to 5) of the menstrual cycle within 60 days of the anniversary visit date (usually because of irregular menstrual cycles), blood was obtained without respect to menstrual bleeding.(24)

For the SWAN bone substudy, five of the SWAN sites (Boston, Pittsburgh, Detoit, Oakland, and Los Angeles, *n* = 2413 at baseline) measured BMD at baseline and annually.(25,26) These sites also collected non-first-voided urine samples (before 0900 hours) between menstrual cycle days 2 and 5 for assay of NTX. Serum and urine samples were frozen within 10 hours of collection and stored at −20 or −80°C for 1 to 60 days at local sites until analysis at a centralized laboratory (Medical Research Laboratories, Highland Heights, KY, USA).

All protocols were approved by the institutional review board at each of the SWAN sites. All participants provided signed, written informed consent.

### Assessment of vasomotor symptoms (VMS)

At baseline and at each annual follow-up visit, women completed a questionnaire that included a symptom checklist. The symptom checklist was worded as follows: “Below is a list of common problems which affect us from time to time in our daily lives. Thinking back over the past two weeks, please circle the number corresponding to how often you experienced any of the following.” Participants rated the frequency of hot flashes and the frequency of night sweats in the past 2 weeks using response choices of never, 1 to 5 days, 6 to 8 days, 9 to 13 days, or daily. For each visit, we classified women who had hot flashes or night sweats or both hot flashes and night sweats as having VMS. For this study, we classified VMS as frequent if they occurred for 6 or more days in the past 2 weeks and not frequent if they occurred for fewer than 6 days (or not at all) in the past 2 weeks.

### Other questionnaire-based and anthropometric measures

At baseline and at each annual follow-up visit, SWAN participants completed standardized questionnaires and underwent measurement of height and weight for calculation of body mass index (BMI, weight in kilograms divided by the square of height in meters). We collected information regarding age, race/ethnicity, reproductive and menstrual history, medication use, smoking, physical activity, dietary intake, and alcohol intake from baseline and annual questionnaires.

Menopausal status, based on self-reported menstrual cycle characteristics recalled over the past year, were premenopausal (menstruation in the past 3 months with no change in menstrual regularity in the past year), early perimenopausal (menstruation in the past 3 months with decreased regularity in the past year), late perimenopausal (no menses for 3 to 11 months), and postmenopausal (no menses for the past 12 months).

At baseline and at annual follow-up visit 5, using a modified 1995 Block Food Frequency Questionnaire, we assessed usual alcohol intake in kilocalories per day.(27–29) Annual questionnaires assessed the usual frequency of supplement intakes of calcium and vitamin D since the last visit using response choices of “Don't take any,” “1 to 3 days per week,” “4 to 6 days per week,” or “every day.”

SWAN used an adaptation of the Kaiser Physical Activity Survey to assess physical activity at baseline, annual follow-up visit 3, and annual follow-up visit 6.(30) Using Likert-scale responses from 1 to 5, the questionnaire asked about several domains of physical activity: household/caregiving, sports/exercise, and active living (walking or biking for transportation, hours of television viewing reverse-scored). The physical activity score was the sum of the active living, sports, and household caregiving scores (range 4 to 20).

### Laboratory assay methodology

At baseline and annually through follow-up visit 8, urinary NTX level was measured using an automated competitive immunoassay (Vitros ICi, Rochester, NY, USA). NTX was expressed as nanomoles of bone collagen equivalents per liter per millimole of creatinine per liter (nM BCE/mM creatinine). The lower limit of detection was 10 nM BCE, and intra- and interassay coefficients of variation were 2.75% and 4.8%, respectively, over the assay range. Creatinine was measured on the Cobas Mira (Horiba ABX, Montpellier, France) based on the Jaffe reaction. The lower limit of detection was 0.014 mM, and the intra- and interassay coefficients of variation were 0.62% and 4.12%, respectively, across the assay range.

To measure serum estradiol level, SWAN used a semiautomated competitive ACS:180 immunoassay with manual steps and an off-line incubation. Inter- and intraassay coefficients of variation averaged 10.6% and 6.4%, respectively, at an estradiol level of 50 pg/mL.(24) Serum follicle-stimulating hormone (FSH) was measured with a two-site chemiluminometric immunoassay using constant amounts of two monoclonal antibodies, each of which was directed at different regions on the β subunit. One antibody was coupled with paramagnetic particles; the other antibody was labeled with dimethylaminoethanol.(24) Inter- and intraassay coefficients of variation were 12.0% and 6.0%, respectively, at an FSH level of 15 IU/L.(24)

### Analytic sample

This analysis is based on data from SWAN bone study participants for whom we had at least one concurrent measurement of NTX and VMS (*n* = 2336 participants at baseline). For this study, we censored data from women at the time they reported having a hysterectomy or bilateral oophorectomy, becoming pregnant, or breast-feeding. We also excluded data from women taking medications that might influence osteoporosis (eg, corticosteroid pills, epilepsy medications, alendronate, risedronate, raloxifene, calcitonin, teriparatide, ibandronate, etidronate, fluoride, chemotherapy, tamoxifen, or exogenous hormone therapy) by censoring their data starting at the time they first reported taking the exclusionary medications. For example, if a participant first reported using an exclusionary medication at annual follow-up visit 6, information for that participant was excluded for visit 6 and all subsequent follow-up visits. These criteria resulted in an analytic sample of 2283 participants at baseline and 686 participants at follow-up 8.

### Statistical analysis

We used repeated-measures mixed models with an autoregressive AR1 model to account for within-woman correlation.(31) We examined the associations between VMS in each of the four menopausal stage categories (the primary predictors) and repeated annual measurement values of urinary NTX (outcome). On the basis of prior studies,(32) we included age (continuous), cigarette smoking (current versus not current), BMI (continuous, kg/m^2^), race/ethnicity (African-American, Caucasian, Chinese, or Japanese), calcium and vitamin D supplement intake (none, 1 to 3 days per week, 4 to 6 days per week, or daily), physical activity score, alcohol intake (kcal/day, log-transformed), and study site as covariates in all multivariable models. For physical activity and alcohol intake, we used the most recent values. All covariates were treated as time-varying covariates except for race/ethnicity and study site.

We conducted analyses using multiple definitions of the VMS outcome to assess robustness and consistency of results. Three sets of models were run, the first set with VMS categorized as any versus none, the second set with VMS categorized as frequent versus not frequent (≥6 days versus <6 days in the past 2 weeks), and the third set with VMS frequency (the higher value of frequency of hot flashes or frequency of night sweats in the past 2 weeks) treated as an ordinal variable ranging from 1 to 5 (1 = never; 2 = 1 to 5 days; 3 = 6 to 8 days; 4 = 9 to 13 days; 5 = daily).

Based on prior analyses of associations between VMS and BMD, we had an a priori hypothesis that associations of VMS with NTX would vary by menopausal stage.(33) Thus we included a VMS × menopausal stage interaction term in the repeated-measures mixed models.

We performed secondary analyses in which we added serum estradiol level and serum FSH level (individually and then simultaneously) as time-varying covariates to the repeated-measures models described earlier. In these models, we included an indicator variable representing whether serum for FSH and estradiol levels was drawn in the early follicular phase of the menstrual cycle (days 2 to 5 versus not days 2 to 5 or unknown). We additionally analyzed associations between VMS and NTX in two separate strata according to whether levels were drawn in the early follicular phase or not.

Statistical analyses were performed using SAS 9.2 (SAS Institute, Inc., Cary, NC, USA).

## Results

### Participant characteristics

In our final analytic sample, the number of participants was 2283 at baseline and 686 at follow-up 8 (Table 1). At baseline, 54% of participants were premenopausal, and 46% of participants were early perimenopausal (Table 2). Mean age at baseline was 45.8 years, and mean BMI was 28.0 kg/m^2^. Forty-nine percent of the analytic sample was white. At baseline, 31.5% of premenopausal participants and 47.2% of early perimenopausal participants reported having any VMS (Table 3). At baseline, frequent VMS (≥6 days in the past 2 weeks) were reported by 7.6% of premenopausal women and 14.7% of early perimenopausal women. By the eighth annual follow-up visit, 44.4% of premenopausal participants, 47.3% of early perimenopausal participants, 72.6% of late perimenopausal participants, and 62.4% of postmenopausal participants reported having any VMS. By the eighth annual follow-up visit, frequent VMS (≥6 days in the past 2 weeks) were reported by 11.1% of premenopausal women, 15.8% of early perimenopausal women, 40.6% of late perimenopausal women, and 29.4% of postmenopausal women.

**Table 1:** Numbers of Participants With Available Urinary *N*-Telopeptide (NTX) Measurement According to Year of Follow-up

	Visit number^a^								
	
	0 = baseline	1	2	3	4	5	6	7	8
Total no. of participants	3302	2881	2746	2710	2679	2577	2448	2368	2279
No. of participants for whom data were available regarding NTX, VMS,^b^ and menopausal stage	2336	2043	1944	1830	1728	1610	1707	1663	1061
No. of participants after further censoring of women with hysterectomy, bilateral oophorectomy, pregnancy, breast-feeding, hormone therapy use, use of medication known to influence bone density, and missed visits^c^	2283	1760	1521	1330	1199	1110	1147	1099	686

a Visit number 0 denotes baseline visit.

b VMS = vasomotor symptoms.

c Medication known to influence bone density includes self-reported use of corticosteroid pills, medications for epilepsy, tamoxifen, chemotherapy, or medications used to prevent or treat osteoporosis. Women were censored at the first visit at which they reporting taking any of these medications.

**Table 2:** Characteristics of Participants at Baseline and at Annual Visits 5 and 8^a^

	Visit number		
	
Characteristic	Baseline (*n* = 2283), mean (SD)	Visit 5 (*n* = 1110), mean (SD)	Visit 8 (*n* = 686), mean (SD)
Body mass index (kg/m^2^)	28.0 (7.5)	28.0 (7.1)	28.2 (6.8)
Physical activity score^b^	7.8 (1.8)	7.7 (1.8)	7.8 (1.8)
Weight	74.2 (21.3)	73.6 (20.0)	74.1 (19.4)
Alcohol, kcal/day	42.7 (96.1)	39.8 (97.1)	40.9 (86.3)
Age, years	45.8 (2.7)	50.9 (2.7)	53.5 (2.5)
Urinary NTX, nM BCE/mM of creatinine	34.4 (16.0)	37.6 (19.4)	43.5 (19.8)
	*n* (percent)	*n* (percent)	*n* (percent)
Smoking, current	370 (16%)	138 (12%)	62 (9%)
Body mass index			
Underweight < 19 kg/m^2^	82 (4%)	37 (3%)	18 (3%)
Normal weight 19–24.9 kg/m^2^	929 (41%)	421 (38%)	237 (35%)
Overweight 25–29.9 kg/m^2^	531 (24%)	299 (27%)	194 (28%)
Obese ≥ 30 kg/m^2^	714 (32%)	352 (32%)	236 (34%)
Ethnicity			
African-American	647 (29%)	269 (25%)	159 (24%)
Caucasian	1127 (49%)	497 (46%)	328 (49%)
Chinese	243 (11%)	149 (14%)	84 (13%)
Japanese	266 (12%)	174 (16%)	98 (15%)
Menopausal stage			
Premenopausal	1230 (54%)	71 (6%)	18 (3%)
Early perimenopausal	1053 (46%)	491 (44%)	184 (27%)
Late perimenopausal	0	155 (14%)	106 (15%)
Postmenopausal	0	393 (35%)	378 (55%)
Vitamin D			
Don't take any	1959 (96%)	1018 (92%)	630 (92%)
1–3 days/week	23 (1%)	16 (1%)	14 (2%)
4–6 days/week	10 (1%)	15 (1%)	12 (2%)
Every day	39 (2%)	56 (5%)	30 (4%)
Calcium supplement use			
Don't take any	1503 (74%)	712 (65%)	469 (68%)
1–3 days/week	136 (7%)	98 (9%)	50 (7%)
4–6 days/week	94 (5%)	69 (6%)	38 (6%)
Every day	297 (15%)	225 (20%)	129 (19%)

a Information regarding these participant characteristics was obtained annually from baseline to annual follow-up visit 8, except for alcohol intake (assessed at baseline and annual visit 5) and physical activity (assessed at baseline, annual visit 3, and annual visit 6). The values displayed for physical activity and for alcohol intake are those from the most recent prior assessment.

b Sum of household/caregiving index, activity living index, and sports index, each scored between 1 and 5, summary physical activity score possible range 3 to 15.

**Table 3:** Menopausal Stage and VMS Reporting at Baseline and at Annual Follow-up Visits 5 and 8^a^

	Visit number								
	
	Baseline			5			8		
			
	No VMS	VMS 1–5 days	VMS 6+ days	No VMS	VMS 1–5 days	VMS 6+ days	No VMS	VMS 1–5 days	VMS 6+ days
Premenopausal	842 (68.5%)	294 (23.9%)	94 (7.6%)	44 (62.0%)	21 (29.6%)	6 (8.5%)	10 (55.6%)	6 (33.3%)	2 (11.1%)
Early perimenopausal	556 (52.8%)	342 (32.5%)	155 (14.7%)	252 (51.3%)	163 (33.2%)	76 (15.5%)	97 (52.7%)	58 (31.5%)	29 (15.8%)
Late perimenopausal	0	0	0	55 (35.5%)	49 (31.6%)	52 (32.9%)	29 (27.4%)	34 (32.1%)	43 (40.6%)
Postmenopausal	0	0	0	169 (43.0%)	106 (27.0%)	118 (30.0%)	142 (37.6%)	125 (33.1%)	111 (29.4%)

a Information regarding VMS (vasomotor symptoms) was obtained annually from baseline to annual follow-up visit 8. Data are presented as *n* (%).

### Association of VMS and NTX according to menopausal stage

A pattern of increasing mean unadjusted urinary NTX level was observed with advancing menopausal stages (Table 4). NTX values were statistically significantly higher among women with VMS (any versus none) than among women who did not have VMS at every menopausal stage except at the premenopausal stage. When NTX values among women with frequent VMS were expressed as percentages of NTX values among women without frequent VMS, the differences were 3% for premenopausal women, 9% for early perimenopausal women, 7% for late perimenopausal women, and 4% for postmenopausal women (calculated from values in Table 4; data not shown).

**Table 4:** Unadjusted Urinary NTX Level According to Presence of VMS by Menopausal Transition Stage^a^

	VMS any versus none^b^				VMS frequent versus not frequent^c^			
		
		NTX level^d^				Ntx level		
						
	VMS	Mean	SD	*p* Value^e^	VMS	Mean	SD	*p* Value^f^
Premenopausal	Overall (*n* = 2500)	33.51	15.45					
	None (*n* = 1739)	33.37	15.64	.48	Not frequent (*n* = 2323)	33.45	15.56	.44
	Any (*n* = 761)	33.84	15.02		Frequent (*n* = 177)	34.38	13.97	
Early perimenopausal	Overall (*n* = 6066)	34.64	16.69					
	None (*n* = 3241)	33.92	15.76	<.001	Not frequent (*n* = 5218)	34.20	16.30	<.001
	Any (*n* = 2825)	35.47	17.66		Frequent (*n* = 848)	37.35	18.70	
Late perimenopausal	Overall (*n* = 1040)	43.30	19.04					
	None (*n* = 324)	42.44	18.22	.33	Not frequent (*n* = 677)	42.22	18.40	.01
	Any (*n* = 716)	43.69	19.40		Frequent (*n* = 363)	45.31	20.05	
Postmenopausal	Overall (*n* = 2529)	47.03	24.97					
	None (*n* = 995)	45.73	28.06	.04	Not frequent (*n* = 1793)	46.53	26.24	.09
	Any (*n* = 1534)	47.88	22.72		Frequent (*n* = 736)	48.26	21.56	

a Values of NTX (nM BCE/mM of creatinine) are averages over all years of follow-up within the given menopausal stage.

b Women were considered to have vasomotor symptoms if they reported experiencing hot flashes or night sweats or both in the past 2 weeks on annual questionnaires.

c “Frequent VMS” indicates VMS frequency ≥ 5 days in the past 2 weeks; “Not frequent VMS” indicates VMS frequency < 5 days in the past 2 weeks.

d Units of NTX are nM BCE/mM of creatinine.

e *p* Value for *t* test comparing mean NTX level of women with any VMS with the mean NTX level of women with no VMS.

f *p* Value for *t* test comparing mean NTX level of women with frequent VMS with the mean NTX level of women without frequent VMS.

The association of VMS with mean urinary NTX varied by menopausal stage (*p*_interaction_ = .07 when VMS was expressed as any versus none; *p*_interaction_ = .01 when VMS was expressed as frequent versus not frequent).

NTX levels were similar among women reporting never having hot flashes compared with women having hot flashes 1 to 5 days in the past 2 weeks (ANOVA *p* > .05). NTX levels likewise were similar among women reporting 6 to 8 days, 9 to 13 days, or daily hot flashes in the past 2 weeks (ANOVA *p* > .05) but different from those among women with no hot flashes or 1 to 5 days of hot flashes in the past 2 weeks (*p* < .05). Thus we do not show results of analyses in which VMS frequency was treated as an ordinal variable for subsequent adjusted analyses.

During the early and late perimenopause, the presence of any VMS was associated with higher urinary NTX values after adjustment for age, current smoking, race/ethnicity, calcium and vitamin D supplement use, physical activity level, alcohol intake, and study site (Table 5). Compared with women in the same menopausal transition stage without VMS, urinary NTX values were 1.94 nM BCE/mM of creatinine higher in early perimenopausal women (*p* < .0001) and 2.44 nM BCE/mM of creatinine higher in late perimenopausal women (*p* = .03) with any VMS.

**Table 5:** Associations Between VMS and Urinary NTX Level in Multivariable-Adjusted Models^a^

	VMS any versus none: basic model			VMS frequent versus not frequent: basic model^b^		
		
Menopausal stage	β coefficient^c^	SD	*p* Value	β coefficient^d^	SD	*p* Value
Premenopausal	1.017	0.809	.17	0.741	1.436	.61
Early perimenopausal	1.944	0.486	<.0001	3.077	0.675	<.0001
Late perimenopausal	2.437	1.011	.03	3.625	1.069	<.001
Postmenopausal	1.259	0.769	.10	1.909	0.799	.02

	VMS any versus none: model with FSH^e^			VMS frequent versus not frequent: model with FSH		
		
Menopausal stage	β coefficient	SD	*p* Value	β coefficient	SD	*p* Value
Premenopausal	0.802	0.805	.32	0.391	1.430	.78
Early perimenopausal	1.347	0.486	.006	2.024	0.676	.003
Late perimenopausal	1.071	1.107	.33	2.519	1.073	.02
Postmenopausal	0.546	0.769	.48	0.919	0.801	.25

	VMS any versusnone: model with estradiol^f^			VMS frequent versus not frequent: model with estradiol		
		
Menopausal stage	β coefficient	SD	*p* Value	β coefficient	SD	*p* Value
Premenopausal	0.969	0.812	.23	0.701	1.442	.63
Early perimenopausal	1.784	0.489	<.001	2.827	0.679	<.0001
Late perimenopausal	2.022	1.113	.07	3.294	1.079	.002
Postmenopausal	1.307	0.774	.09	1.748	0.805	.06

	VMS any versus none: model with FSH and estradiol^g^			VMS frequent versus not frequent: model with FSH and estradiol		
		
Menopausal stage	β coefficient	SD	*p* Value	β coefficient	SD	*p* Value
Premenopausal	0.787	0.805	.33	0.443	1.430	.76
Early perimenopausal	1.336	0.486	.006	1.022	0.676	.003
Late perimenopausal	1.042	1.107	.35	2.492	1.073	.02
Postmenopausal	0.557	0.769	.47	0.930	0.801	.25

a Repeated-measures models adjusted for age, smoking, BMI, race/ethnicity, calcium and vitamin D supplement intake, physical activity score (log-transformed), alcohol intake (kcal/d, log-transformed), and study site.

b “Frequent VMS” indicates VMS frequency ≥ 5 days in the past 2 weeks; “Not frequent VMS” indicates VMS frequency < 5 days in the past 2 weeks.

c β coefficient represents the increment in urinary NTX (nM BCE/mM of creatinine) associated with reporting any VMS versus no VMS.

d β coefficient represents the increment in urinary NTX (nM BCE/mM of creatinine) comparing frequent VMS with not frequent VMS.

e Includes variables of basic model, follicle-stimulating hormone level (FSH, IU/L) and menstrual cycle phase (follicular versus not follicular or unknown phase).

f Repeated-measures models adjusted for variables of the basic model, serum estradiol concentration (pg/mL), and menstrual cycle phase.

g Repeated-measures models adjusted for variables of the basic model, serum FSH level, serum estradiol concentration, and menstrual cycle phase.

Compared with women in the same menopausal transition stage without frequent VMS, urinary NTX values were 3.08 nM BCE/mM of creatinine higher in early perimenopausal women (*p* < .0001), 3.63 nM BCE/mM of creatinine (*p* < .001) higher in late perimenopausal women, and 1.91 nM BCE/mM of creatinine higher in postmenopausal women (*p* = .02) with VMS occurring frequently (≥6 days in past 2 weeks). Neither the presence nor the frequency of VMS was significantly associated with NTX level among premenopausal women. When VMS frequency was treated as an ordinal variable ranging 1 to 5 (1 = none; 2 = 1 to 5 days; 3 = 6 to 8 days; 4 = 9 to 13 days; 5 = daily in the past 2 weeks), the pattern and statistical significance of associations were similar to the results of the previous models (data not shown).

### Secondary analyses adjusted for serum estradiol and FSH levels

Addition of FSH level to the multivariable repeated-measures regression models markedly decreased the magnitude of but did not completely eliminate associations between VMS and NTX level, whereas adjustment for serum estradiol level had a lesser impact on the magnitudes of associations between VMS and NTX level (Table 5). Simultaneous adjustment for estradiol and FSH produced results that were virtually identical to adjustment for FSH level alone.

When we repeated mixed-effects regression models among women whose FSH and estradiol levels were not drawn during the early follicular phase (53% of estradiol and FSH values), the magnitudes of associations between VMS and NTX were more pronounced but showed a similar pattern of statistical significance compared with the analytic sample as a whole (data not shown). In contrast, among women whose estradiol and FSH levels were drawn in the early follicular phase, no statistically significant associations were observed between VMS and NTX.

## Discussion

In this longitudinal study, we found VMS to be positively associated with NTX level among perimenopausal and postmenopausal women. These associations were in large part decreased by adjustment for serum FSH level but not by adjustment for estradiol level. The pattern of results was similar regardless of whether VMS frequency was characterized as any versus none, as frequent versus not frequent, or as numbers of days of VMS in the past 2 weeks.

Although the longitudinal associations of NTX and BMD have not been established among women undergoing the menopause transition, randomized, controlled trials of osteoporosis pharmacotherapy in older women have related changes in NTX level to changes in BMD. For example, a 30% decrease in NTX level after 6 months of alendronate therapy corresponded to a 5.8% increase in lumbar BMD after 2.5 years of therapy.(34) The relations between NTX and BMD based on clinical trials cannot be extrapolated directly to observational studies; however, they suggest that the 9% difference in NTX between early perimenopausal women with VMS and early perimenopausal women without VMS may correspond to differences in BMD of 2%.

We are aware of only one previous study that has focused on associations between VMS and urinary NTX level.(22) That cross-sectional study of premenopausal infertile women found serum NTX to be higher among women reporting having night sweats than among women reporting not having night sweats but found no significant association between hot flashes and NTX level.(22) Because we did not find statistically significant associations between VMS and NTX among the premenopausal women, our results are in agreement with that prior study. Moreover, our longitudinal study design may have allowed enhanced sensitivity to detect associations of VMS and urinary NTX.

These results may extend prior studies that reported lower BMD among midlife women with VMS.(8–11) Using data from SWAN, we previously found that women with VMS had lower BMD on average than women without VMS.(9) Our findings support the hypothesis that elevated NTX is one mechanism for the inverse association of VMS with BMD among midlife women.

Our findings are biologically plausible. We have already explored one possible biologic explanation for our findings in this study: Serum FSH level appeared in part to explain the associations between VMS and NTX level. In prior studies, serum FSH concentration was associated with both higher NTX level and higher frequency of VMS, and longitudinal losses of BMD were more strongly associated with increases in FSH levels rather than with changes in estradiol levels, albeit some of these studies are similarly constrained by early-follicular-phase sampling.(24,35–39) Although others have found serum estradiol level to be associated with BMD over the menopause transition.(40) adjustment for serum total estradiol did not influence the associations between VMS and NTX in our study. In a cross-sectional analysis, elevated bone turnover was apparent among perimenopausal women despite circulating estradiol levels that were similar to those of premenopausal women.(39)

Prior to the final menstrual period, changes in gonadal inhibins may cause the trabecular bone loss that has been observed prior to the onset of menopause.(41) The rise in FSH during the menopause transition is preceded by declining levels of inhibin B, reflecting the fall in ovarian follicle levels, whereas estradiol levels remain largely unchanged or rise with age until late perimenopause, when FSH levels increase.(42) FSH may have direct effects on bone in part by enhancing receptor activator of nuclear factor κB ligand (RANKL)–stimulated osteoclast development and activity.(43) Inhibins likely inhibit osteoblastogenesis and osteoclastogenesis (reviewed in ref. (44)). In a cross-sectional study, FSH was significantly correlated with bone resorption among perimenopausal women, but serum inhibin A predicted bone resorption levels better in pre- and perimenopausal women than did FSH or bioavailable estradiol.(45)

Besides circulating FSH and inhibin levels, other possible biologic mechanisms may underlie our findings. Although associations of cortisol with increased urinary NTX among the general population of midlife women have not been the focus of previous studies, elevated cortisol levels are associated with decreased BMD in certain disease states, such as major depression and Cushing syndrome.(46,47) Because cortisol levels increase in the late perimenopausal stage,(48) and because cortisol levels may increase acutely after hot flashes,(12,14,49) increased cortisol levels associated with frequent VMS also may lead to elevated NTX level. Although we were not able to test this hypothesis directly owing to the lack of availability of data regarding cortisol levels, we will explore possible mechanisms underlying associations of VMS with NTX level.

In stratified analyses, no association between VMS and NTX was evident among the subsample of participants whose serum estradiol and FSH levels were drawn in the early follicular phase. Thus the associations observed in the full sample were being driven by the women whose blood samples were drawn outside the early follicular phase, that is, in late perimenopausal and postmenopausal women. The inability to detect associations between VMS and NTX among women who had early-follicular-phase phlebotomy may be explained by the low variation in VMS and NTX among women with early-follicular-phase estradiol and FSH sampling (who were more likely to be pre- or early perimenopausal women) compared with women whose blood samples were not drawn in the early follicular phase (who were more likely to be late peri- or postmenopausal). In this study, it is not possible to completely disaggregate menopausal stage per se from timing (early follicular phase versus not early follicular phase) of blood sampling.

Finally, another explanation for the associations of VMS with NTX level may be that within a given menstrually based menopause stage, VMS are an indication of more advanced ovarian aging (ie, that when added to menstrually based menopause transition stage, VMS further refine the estimation of ovarian aging). We would expect that a more advanced menopausal stage would be associated with higher bone turnover.

A previous SWAN analysis demonstrated that BMD changed little during pre- or early perimenopause but declined substantially in the late perimenopausal period.(50) The rate of BMD loss in late perimenopause was similar to that in the first two postmenopausal years.(50) A time lag may occur in the BMD response to increasing NTX levels so that the early perimenopausal rise in NTX would be apparent before the late perimenopausal drop in BMD noted in the prior study.

Strengths of our study include its community-based multiethnic recruitment strategy, large sample size, and longitudinal design. Our study had potential limitations. First, our method of VMS assessment, which has been used frequently in longitudinal studies, theoretically could have yielded an underestimate of true VMS frequency, that is, would not have assessed VMS frequency other than in the past 2 weeks. Thus our results may have been attenuated as a result of this type of misclassification. Also, we did not include measures of daily frequency and bothersomeness of hot flashes because these were not obtained in SWAN until visit 7; thus future SWAN analyses with more longitudinal data should examine the relation of these measures to bone turnover. Second, our annual questionnaire assessments of calcium intake, vitamin D intake, and physical activity levels may not have been as reliable as those which would have been afforded by prospectively collected food and supplement diaries, which might have resulted in a conservative bias (toward the null).

In conclusion, among early perimenopausal and late perimenopausal women, we found that women with VMS had significantly higher NTX levels than women without VMS. Prior to the final menstrual period, VMS may be a marker for elevated bone turnover. It is not definitely established whether women with VMS prior to the final menstrual period are at higher risk of osteoporotic fracture. However, high bone turnover is associated with lower BMD, faster bone loss, and poor bone microarchitecture both in the trabecular compartment (trabecular perforations, loss of trabeculae, poor trabecular connectivity) and in the cortical compartment (cortical thinning, increased porosity).(51–53)
